# Establishment of the Relationship between Tumor Size and Range of Histological Involvement to Evaluate the Rationality of Current Retinoblastoma Management

**DOI:** 10.1371/journal.pone.0080484

**Published:** 2013-11-28

**Authors:** Jianhua Yan, Hao Zhang, Yongping Li

**Affiliations:** The State Key Laboratory of Ophthalmology, Zhongshan Ophthalmic Center, Sun Yat-sen University, Guangzhou, The People’s Republic of China; Massachusetts Eye & Ear Infirmary, Harvard Medical School, United States of America

## Abstract

**Purpose:**

To determine whether tumor size correlates with histopathological involvement and hence evaluate the rationality of conservative treatment for retinoblastoma.

**Methods:**

We retrospectively studied 221 patients (221 eyes) treated for retinoblastoma with enucleation in the Zhongshan Ophthalmic Center of Sun Yat-sen University, China, from October 1995 to December 2004. Histopathological data included involvement of the anterior chamber, sclera, choroids, and optic nerve. Tumor size was measured by B-ultrasound examination.

**Results:**

Tumor invasion of the optic nerve correlated with the Reese-Ellsworth (R-E) staging system and the International Classification for Retinoblastoma (ICRB): optic nerve involvement was significantly more frequent in R-E stage V (P = 0.009) and ICRB Group E (P = 0.002) cases. However, 19.1% of patients with R-E stage I, II and III, and 16.7% of patients with ICRB Group B and C disease showed histopathological involvement of the postlaminar optic nerve. Extraocular involvement was observed in 17.7% of tumors ≤15 mm in diameter. Tumors >15 mm in diameter showed greater extraocular involvement, including the optic nerve (P = 0.000) and sclera (P = 0.032), than tumors ≤15 mm in diameter. Postlaminar optic nerve invasion was observed in 19.6% of tumors ≤10 mm in thickness. Tumors >10 mm in thickness had sclera involvement more frequently than tumors ≤10 mm in thickness (P = 0.029). Postlaminar optic nerve invasion was noted in 17.1% of patients with tumors ≤15 mm in diameter and ≤10 mm in thickness.

**Conclusions:**

Medium-sized retinoblastomas frequently invade outside the globe. Thus, indications for conservative treatment need improvement.

## Introduction

Retinoblastoma is the most common malignant intraocular tumor in children. An overall survival rate of 90–95% can now be achieved because of earlier diagnosis and improved methods of treatment [1], [2]. In developed countries, it is estimated that currently more than 50% of children with retinoblastoma are managed initially with chemoreduction, in an effort to avoid radiotherapy or enucleation [3], [4]. Chemoreduction plus focal consolidation treatment has become an important therapeutic approach for retinoblastoma. Increasing numbers of patients with large retinoblastoma can be managed with conservative treatment such as systemic chemotherapy [2]. In the last few years, intra-arterial chemotherapy has replaced chemoreduction and has emerged as a promising treatment alternative for early or advanced retinoblastomas in many centers worldwide. However, in developing countries such as China a relatively high percentage of patients with retinoblastoma are still managed with enucleation, because of the large tumor size or the advanced stage of the disease. A small proportion of children with tumor sizes of 8–15 mm in basal dimension and 5–10 mm in thickness also have to undergo enucleation because of the financial burden of globe-sparing treatment modalities, although most of these patients would be treated with conservative methods in developed countries. We found that histopathologic examination of some of these children revealed postlaminar optic nerve invasion. Retinoblastoma patients with optic nerve invasion should be managed with enucleation [1]. However, the percentage of patients with this size of tumor who have optic nerve invasion is not clear. Accordingly, whether the current established criteria for choosing conservative therapeutic methods for retinoblastoma are accurate is not established. Therefore, we decided to analyze the relationship between tumor size (clinical measurement) and range of involvement (histopathologic examination) in order to evaluate the rationality of the current management of retinoblastoma.

## Materials and Methods

Three hundred and fifty consecutive patients with retinoblastoma, who were referred to Zhongshan Ophthalmic Center, Sun Yat-sen University, the People’s Republic of China, from October 1995 to December 2004, were evaluated. The ethics committee of Zhongshan Ophthalmic Center approved this retrospective study and our paper has been conducted according to the principles expressed in the Declaration of Helsinki. The committee specifically waived the need for consent. All patients were Chinese. Among them, 221 patients met the inclusion criteria for this study. Our inclusion criteria encompassed all those patients with retinoblastoma who underwent enucleation without any prior local or systemic treatment. All diagnoses were confirmed by histopathological examination. The data collected in this study included the patient’s age, sex, laterality, and size and extent of tumor. The retinoblastoma was staged prior to surgery according to the Reese-Ellsworth (R-E) staging system, which has been the gold standard for intraocular staging for decades. The International Classification for Retinoblastoma (ICRB) was also used for our patients. Attention was focused on both tumor size and histopathological involvement of ocular tissues. Eyes with more than 1 tumor were excluded from this study. Only patients with retinoblastoma tumor, rather than multiple types of malignancies were included in this study.

The tumor size in basal dimension (mm) and thickness (mm) was measured using B-ultrasound examination (Au4 Idea, Italy). Tumors from all enucleated patients were examined by our pathologist, and all slides were analyzed immediately after enucleation. The pathologist was blinded to the clinical characteristics of the patients. The optic nerve stump length was measured on pathological slides. Tumor extension within the choroids (focal and massive), sclera (intrascleral and extrascleral or microscopic orbital involvement), and optic nerve (prelaminar involvement, postlaminar involvement without invasion of the optic nerve resection line, and postlaminar involvement with invasion of the resection line) was determined. Choroidal involvement was characterized as massive when more than one-fourth of the choroids were invaded by the tumor. Involvement of the anterior chamber was also examined. Histopathological slides from all patients with retinoblastoma in this study were carefully re-examined by the same pathologist (Y Li) using the same criteria, although some possibility of missed choroidal, extrascleral, or optic nerve invasion may exist because of selection bias. In all patients, 1 representative plane through the globe was examined, and this section contained the pupil, the optic nerve, and the maximal elevation of the tumor. Additional cross-sections through the distal end of the optic nerve, taken before the sectioning of the eye, were also routinely performed.

Statistical analysis was performed using SPSS version 17.0 (SPSS Inc, Chicago, IL). P-values of less than or equal to 0.05 was considered statistically significant. We compared the clinical data of the 2 groups by using the Pearson Chi square, Yates’ correction and Fisher’s Exact test.

## Results

Among the 221 patients, 138 were male and 83 were female, with an average age of 29.6 months. Leukocoria was the most common presenting symptom. There were 202 unilateral cases and 19 bilateral cases. Two hundred and twenty-one eyes underwent enucleation, and a segment (average of 12 mm, range 8–15 mm) of the optic nerve was removed to avoid the possibility of a residual tumor in the optic nerve stump. There were only 8 eyes with more than 1 intraocular tumor, and they were excluded from this study because of the small number of cases.

**Figure 1:**
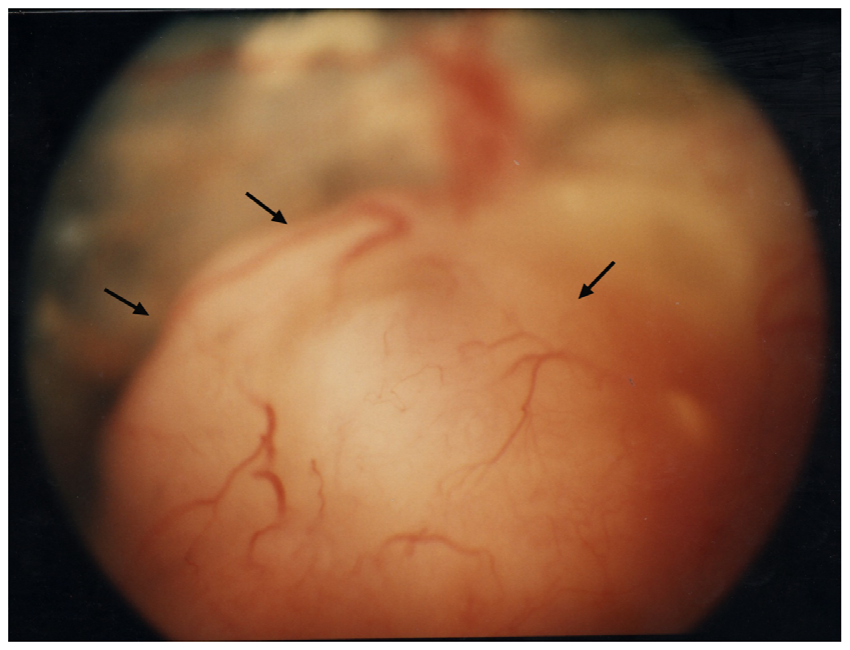
Photograph of the fundus showing a pink-white tumor of the right retina in a 2-year-old child, classified as Reese-Ellsworth classification II and International Classification for Retinoblastoma Group C.

**Figure 2:**
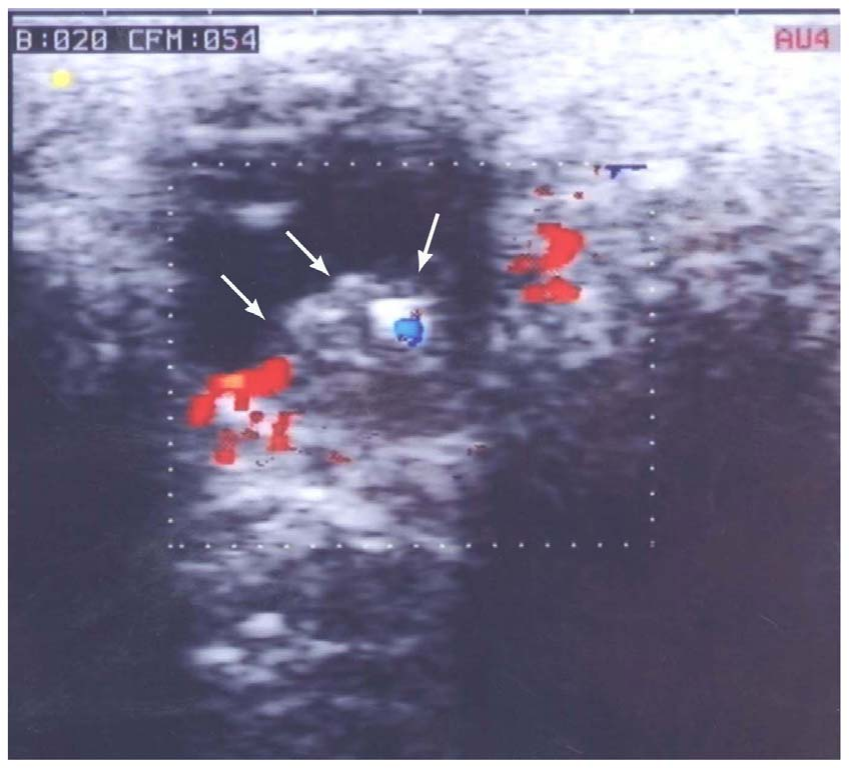
Color Doppler ultrasound examination showing an intraocular tumor, 8× 6 mm, with rich blood flow within the mass.

**Figure 3:**
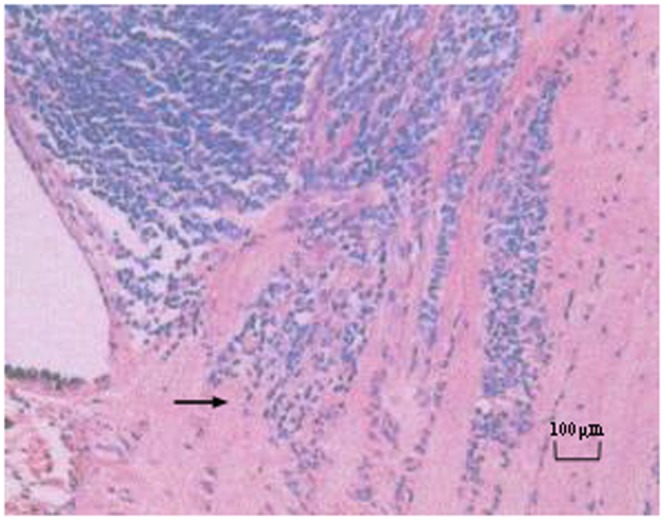
Histopathology slides showing postlaminar optic nerve invasion of the tumor cells (hematoxylin-eosin stain).

Histopathological outcomes of retinoblastoma according to R-E classification are listed in Table 1. Patients with R-E classification I, II, III, IV, and V showed no statistically significant difference in the histopathological involvement of the anterior chamber (P = 0.912), sclera (P = 0.219), or choroids (P = 0.220). However, histopathological involvement of the optic nerve was 50% (2/4), 51.5% (17/33), 67.7% (21/31), 66.7% (18/27), and 81.0% (102/126) in patients with an R-E classification of I, II, III, IV, and V, respectively, and these differences were statistically significant (P = 0.009). It is important to note that 19.1% (13/68) of patients with an R-E classification of I, II, and III showed histopathological involvement of the postlaminar optic nerve (Figures 1–3).

**Table 1:** Histopathological involvements of retinoblastoma using the Reese-Ellsworth Staging systems.

Histopathological involvements	Stage I(4 eyes)	Stage II(33 eyes)	Stage III(31 eyes)	Stage IV(27 eyes)	Stage V(126 eyes)	P value
Anterior chamber	1(25%)	3(9.1%)	3(9.7%)	3(11.1%)	14(11.1%)	0.912
Sclera	0(0%)	1(3.0%)	2(6.5%)	1(3.7%)	17(13.5%)	0.219
Choroid	3(75%)	16(48.5%)	14(45.2%)	14(51.9%)	80(63.5%)	0.220
Focal	3(75%)	16(48.5%)	14(45.2%)	13(48.1%)	69(54.8%)	
Massive	0(0%)	0(0%)	0(0%)	1(3.7%)	11(8.7%)	
Optic nerve	2(50%)	17(51.5%)	21(67.7%)	18(66.7%)	102(81.0%)	0.009
Prelaminar	2(50%)	11(33.3%)	14(45.2%)	13(48.1%)	51(40.5%)	
Postlaminar 1	0(0%)	5(15.2%)	5(16.1%)	4(14.8%)	35(27.8%)	
Postlaminar 2	0(0%)	1(3.0%)	2(6.5%)	1(3.7%)	16(12.7%)	

Postlaminar 1: postlaminar without invasion of cut end of optic nerve;

Postlaminar 2: postlaminar with invasion of cut end of optic nerve.

Histopathological outcomes of retinoblastoma according to the ICRB are listed in Table 2. Patients with ICRB Group A, B, C, D, and E showed no statistically significant difference in histopathological involvement of the anterior chamber (P = 0.379), sclera (P = 0.631), or choroids (P = 0.089). However, histopathological involvement of the optic nerve was 0% (0/0), 37.5% (3/8), 60% (6/10), 57.4% (27/47), and 79.5% (124/156) in patients with ICRB Group A, B, C, D, and E, respectively, and these differences were statistically significant (P = 0.002). It is important to note that 16.7% (3/18) of patients with ICRB Group B and C showed histopathological involvement of the postlaminar optic nerve.

**Table 2:** Histopathological involvements using the International Classification for Retinoblastoma (ICRB).

Histopathological Involvements	Group A (0 eyes)	Group B(8 eyes)	Group C(10 eyes)	Group D(47 eyes)	Group E(156 eyes)	P value
Anterior chamber	0(0%)	0(0%)	0(0%)	4(8.5%)	20(12.8%)	0.379
Sclera	0(0%)	0(0%)	1(10%)	3(6.4%)	17(10.9%)	0.631
Choroid	0(0%)	3(37.5%)	5(50%)	21(44.7%)	98(62.8%)	0.089
Focal	0(0%)	3(37.5%)	4(40%)	18(38.3%)	90(57.7%)	
Massive	0(0%)	0(0%)	1(10%)	3(6.4%)	8(5.1%)	
Optic nerve	0(0%)	3(37.5%)	6(60%)	27(57.4%)	124(79.5%)	0.002
Prelaminar	0(0%)	2(25%)	4(40%)	19(40.4%)	66(42.3%)	
Postlaminar 1	0(0%)	1(12.5%)	1(10%)	5(10.6%)	42(26.9%)	
Postlaminar 2	0(0%)	0(0%)	1(10%)	3(6.4%)	16(10.3%)	

Histopathological outcomes of retinoblastoma according to tumor size are summarized in Table 3. Tumors with a basal dimension of >15 mm did not differ from those with a basal dimension of ≤15 mm with regard to the rate of histopathological involvement of the anterior chamber (P = 0.920) and choroids (P = 0.352). However, the rate of sclera and optic nerve involvement was 12.7% (17/134) and 81.3% (109/134), respectively, in the former group and 3.8% (3/79) and 54.4% (43/79), respectively, in the latter group, and these differences were statistically significant (P = 0.032 and P = 0.000, respectively). Retinoblastoma invasion outside the globe occurred in 17.7% (14/79) of the 79 cases with tumors ≤ 15 mm in basal dimension and in 41.8% (56/134) of the 134 cases with tumors>15 mm in basal dimension. This difference was statistically significant (P = 0.001). Overall, there were 70 eyes for which invasion outside the globe was observed, accounting for 32.9% of the 213 eyes.

**Table 3:** Histopathological involvements of retinoblastoma using different tumor size.

Histopathological Involvements	Tumor size		P value	Tumor thickness		P value
	Diameter>15 mm(134 eyes)	Diameter≤15 mm(79 eyes)		Thickness>10 mm(167 eyes)	Thickness≤10 mm(46 eyes)	
Anterior chamber	13(9.7%)	8(10.1%)	0.920	19(11.4%)	2(4.3%)	0.256
Sclera	17(12.7%)	3(3.8%)	0.032	20(12.0%)	0(0%)	0.029
Intra-sclera	12(9.0%)	2(2.5%)		14(8.4%)	0(0%)	
Extra-sclera	5(3.7%)	1(1.3%)		6(3.6%)	0(0%)	
Choroid	80(59.7%)	42(53.2%)	0.352	98(58.7%)	24(52.2%)	0.429
Focal	71(53.0%)	39(49.4%)		88(52.7%)	22(47.8%)	
Massive	9(6.7%)	3(3.8%)		10(6.0%)	2(4.3%)	
Optic nerve	109(81.3%)	43(54.4%)	0.000	124(74.3%)	28(60.9%)	0.075
Prelaminar	58(43.3%)	30(38.0%)		69(41.3%)	19(41.3%)	
Postlaminar 1	35(26.1%)	11(13.9%)		39(23.4%)	7(15.2%)	
Postlaminar 2	16(11.9%)	2(2.5%)		16(9.6%)	2(4.3%)	

Tumors of >10 mm in thickness did not differ from those of ≤10 mm in thickness with regard to the rate of histopathological involvement of the anterior chamber (P = 0.256), choroids (P = 0.429), or optic nerve (P = 0.075). However, the rate of sclera involvement was 12.0% (20/167) in the former group and 0% (0/46) in the latter group, and this difference was statistically significant (P = 0.029). Retinoblastoma invasion outside the globe occurred in 19.6% (9/46) of the 46 cases in which thickness of the tumors was ≤10 mm and in 36.5% (61/167) of the 167 cases in which thickness of the tumors was>10 mm. This difference was statistically significant (P = 0.030).

Looking further into the post-laminar cut end involvement data, we found that tumors of >15 mm in basal dimension had more postlaminar cut end involvement (11.9%,16/134) than those of ≤15 mm in basal dimension (2.5%, 2/79) (P = 0.017). However, Tumors of >10 mm in thickness did not differ from those of ≤10 mm in thickness with regard to the rate of histopathological involvement of the postlaminar cut end involvement, with the former group being 9.6% (16/167) and the latter group being 4.3% (2/46) (P = 0.406).

In patients with tumors ≤15 mm in basal dimension and ≤10 mm in thickness, the rates of anterior chamber involvement, sclera involvement, choroid involvement, postlaminar involvement without invasion of the cut end of the optic nerve, and postlaminar involvement with invasion of the cut end of the optic nerve were 5.7% (2/35), 0, 57.1% (20/35), 14.3% (5/35), and 2.9% (1/35), respectively. Retinoblastoma invasion outside the globe occurred in 17.1% of cases (6/35).

## Discussion

During the past decade, management of retinoblastoma has moved from enucleation or external beam therapy to conservative therapies, in an attempt to preserve visual acuity and the eyeballs of patients [1], [2], [3], [4], [5], [6], [7], [8]. Several of these management strategies, in particularly chemoreduction, have been proven to provide patients with improved ocular prognosis. With chemoreduction, control of the tumor depends on the R-E stage. In cases of R-E stages I–IV, tumor control of approximately 90% is possible with globe salvage. In cases of R-E stage V, globe salvage enables tumor control of only approximately 50% [9], [10]. Intra-arterial chemotherapy with single-agent injection into the ophthalmic artery has recently become more widely used as a treatment for retinoblastoma. This technique can be useful for eyes that fail standard treatments, for saving eyes with extensive intraocular retinoblastoma scheduled for enucleation, and even for less advanced retinoblastoma as a primary treatment [11], [12], [13]. Recently, Shields et al evaluated the reliability of the ICRB for predicting treatment success with chemoreduction, and treatment success was achieved in more than 90% of patients with Group A, B, and C disease [14]. Several factors need to be taken into account when determining the treatment plan, such as the patient’s visual outcome, age, laterality, the size and position of the tumor, and the presence of sub-retinal or vitreous seeds. Of these factors, the size of tumor is the most important for selection of the treatment method. In general, if a tumor is <15 mm in basal dimension, the eye is likely to be saved. Histopathological examination plays an important role in assessing the prognosis of patients, and several risk factors have been identified [15], [16], [17], [18]. It is generally agreed that optic nerve invasion and extrascleral involvement are strongly predictive of poor prognosis [16], [17], [18], [19], [20]. The simultaneous presence of optic nerve invasion and choroidal invasion is also one of the highest risk factors for metastasis [15], [16], [17], [18], [19], [20], [21]. Comparison between tumor size and the range of histopathologic involvement would allow us to know more about whether our methods of management, which are based mainly on tumor size, are suitable for children with retinoblastoma.

Both the R-E staging system and the ICRB correlated well with histological involvement of the optic nerve, in that, patients with the R-E stage V and/or ICRB Group E disease showed optic nerve involvement more frequently. However, this observation was not significant for histological invasion of the anterior chamber, sclera, or choroids. Extraocular involvement, including involvement of the optic nerve and sclera, was more frequent for tumors >15 mm in diameter than for those ≤15 mm in diameter. In our series, tumor >10 mm in thickness showed sclera involvement more frequently than tumors ≤10 mm in thickness. In our study, extraocular invasion was observed in 32.9% of patients, a rate similar to that reported by Magramm (29.5%) [22] and Shields (29%) [19]. Local treatments such as laser therapy, cryotherapy, and thermotherapy are used primarily for small tumors that are <5 mm in diameter and <3 mm in thickness. Our data showed that no patient with a similarly sized tumor had choroidal and/or optic nerve invasion. Therefore, it is rational to use the current local treatment modalities for patients with small intraocular tumors. Eyes with large tumors occupying more than 50% of the intraocular volume (diameter > 15 mm or thickness > 10 mm), secondary glaucoma, anterior chamber involvement, and optic nerve or orbital tumor extension are usually managed with enucleation. In our study, tumors >15 mm in diameter or >10 mm in thickness showed histopathological invasion of eye tissues to a greater extent, with the extraocular invasion rate being 41.8% (56/134) and 36.5% (61/167), respectively. When only the postlaminar cut end involvement was calculated, as suggested by Shields et al.[19], tumors of >15 mm in basal dimension had more postlaminar cut end involvement (11.9%) than those of ≤15 mm in basal dimension (2.5%). Therefore, it is also rational to use enucleation for these patients with large tumors. Obviously, one very important clinical feature for selecting enucleation versus conservative treatment is the location of the tumor. If the tumor obliterates the optic nerve, enucleation may be strongly indicated, regardless of the tumor size. In addition, sub-retinal seeds and extensive vitreous seeding away from the main mass are indications for external beam radiation or enucleation.

More attention should be paid to patients with medium-sized tumors with a basal dimension of 6–15 mm and a thickness of 4–10 mm. Currently, chemoreduction plus local treatments such as plaque radiotherapy (brachytherapy) are commonly used for these tumors, on the basis of the assumption that the tumors are limited to within the globes. However, recurrence rates of 12–50% have been observed with these methods [1], [10]. We showed that retinoblastoma invasion outside the globe occurred in 17.7% of the 79 cases of tumors <15 mm in basal dimension, in 19.6% of the 46 cases of tumors <10 mm in thickness, and in 17.1% of the 35 cases satisfying both these criteria. Therefore, whether it is rational to use the current conservative methods (i.e., non-enucleation) to treat patients with medium-sized tumors is worthy of discussion.

Our results showed that 19.1% of patients with R-E I, II, and III tumors and 16.7% of patients with ICRB Group B and C tumors showed post-laminar optic nerve invasion. However, cumulative clinical experience in the ocular oncology field over the last 30 years has shown a nearly 100% cure rate for patients harboring early-stage retinoblastoma tumors. However, the nearly 100% cure rate of the patients represents the overall 5-year survival rate only. In the 1990s, a success rate of only 78.5% was reported, even with external beam irradiation in patients with R-E I and II tumors [23]. In the largest report specifically examining patients with R-E I-III tumors treated with primary external beam irradiation, only 85% of the eyes were salvaged and 4% of patients died of metastatic retinoblastoma [24]. Gündüz et al. noted that 23% of R-E I–III tumors required both chemotherapy and subsequent external beam irradiation to salvage the eye [25]. Finally, a recent report from Korea also suggested that only 86% of eyes with R-E I–III tumors were salvaged when 13 cycles of multi-agent systemic chemotherapy were used [26]. Therefore, we should consider the possibility of post-laminar invasion even in patients with R-E I, II, and III tumors or ICRB Group B and C tumors.

The limitations of this study were that our sample size was relatively small, and ours was a retrospective analysis. In clinical practice, before globe-sparing treatment is implemented, an attempt is often made to rule out choroidal invasion, optic nerve invasion, scleral invasion, and anterior segment involvement/neovascular glaucoma using clinical examination, ultrasound examination, and other imaging techniques. However, there are bound to be some cases of retinoblastoma that display high-risk features histologically but do not show clinical manifestations, and these cases are of greatest interest. In the present study, cases in which these high-risk features were clinically suspected were not excluded because all these cases were treated with enucleation. This study successfully correlated tumor size with histopathological involvement and revealed the shortcomings of the current established criteria for the selection of conservative treatment for retinoblastoma.

In conclusion, a higher number of ocular tissues were involved, and the extent of involvement increased with increasing tumor size. Medium-sized retinoblastoma invasion outside the globe occurs at a relatively high incidence, and therefore, the indications for conservative therapies in these patients should be adjusted appropriately. Medium-sized tumors must be followed-up very carefully after conservative therapy, because they may be resistant to therapy and/or treatment might not be sufficient to kill the invasive component that is not manifested clinically. The important questions of “What are the optimal indications for chemotherapy?” and “How do we confirm that the tumor is limited to the eyeball (due to the current limitations in imaging studies to detect invasion into the choroids and optic nerve)?” still remain to be answered.
